# The endosperm microstructure, physical, thermal properties and specific milling energy of spelt (*Triticum aestivum* ssp. *spelta*) grain and flour

**DOI:** 10.1038/s41598-023-30285-9

**Published:** 2023-03-03

**Authors:** Małgorzata Warechowska, Andrzej Anders, Józef Warechowski, Mirosław Bramowicz, Agnieszka Markowska-Mendik, Wojciech Rejmer, Józef Tyburski, Sławomir Kulesza

**Affiliations:** 1 Faculty of Technical Sciences, University of Warmia and Mazury in Olsztyn, Oczapowskiego 11, 10-719 Olsztyn, Poland; 2 Faculty of Food Sciences, University of Warmia and Mazury in Olsztyn, ul. Oczapowskiego 7, 10-719 Olsztyn, Poland; 3 Faculty of Agriculture and Forestry, University of Warmia and Mazury in Olsztyn, Pl. Łódzki 3, 10-719 Olsztyn, Poland

**Keywords:** Mechanical properties, Biomaterials, Scanning electron microscopy

## Abstract

Previous research has shown that the endosperm microstructure and physical properties of grain have significance in grain processing and in the development of processing machines. The aim of our study was to analyze the endosperm microstructure, physical, thermal properties, and specific milling energy of organic spelt (*Triticum aestivum* ssp*. spelta*) grain and flour. Image analysis combined with fractal analysis was used to describe the microstructural differences of the endosperm of spelt grain. The endosperm morphology of spelt kernels was monofractal, isotropic, and complex. A higher proportion of Type-A starch granules resulted in an increased proportion of voids and interphase boundaries in the endosperm. Changes in the fractal dimension were correlated with kernel hardness, specific milling energy, the particle size distribution of flour, and the starch damage rate. Spelt cultivars varied in size and shape of the kernels. Kernel hardness was a property that differentiated specific milling energy, particle size distribution of flour, and starch damage rate. Fractal analysis may be considered as a useful tool for evaluating milling processes in the future.

## Introduction

Spelt wheat (*Triticum aestivum* ssp. *spelta*) is an ancient subspecies of common wheat (Triticum aestivum ssp. aestivum)^1,2^. Grown primarily on organic farms in Europe, spelt wheat is currently experiencing a resurgence in popularity. This is due to increased awareness of the health benefits of organic foods and the environmental benefits of organic farming^3^. In addition to its positive impact on the environment, spelt is also sought after for its unique taste and nutritional benefits. Compared to common wheat, spelt produces seeds with higher protein, lipid, and macro and micronutrient content^2,4,5^. As a result, spelt grain is used in the production of bread, cookies, pasta, beer, and as a raw material to enrich bakery products^2^. While spelt has many desirable characteristics, it also has certain unfavorable properties. One such property is the hard husk that surrounds the spelt seeds, which can be challenging to separate^6,7^. Due to its non-threshable hulls, spelt is more difficult to process, which can cause damage to the grain. A high proportion of damaged grain reduces its technological suitability and market value. A comprehensive understanding of the geometric properties, microstructure, and mechanical properties of cereal grain is essential for designing processing operations and grain processing machines. The cellular structure plays a critical role in determining the mechanical properties of the grain, and different cell arrangements at the microscale will exhibit varied physical responses to external forces. During milling, the grain structure is disrupted, and the specific energy consumed during milling, as well as the final product quality, depend on the mechanical resistance of the grain, the texture of the endosperm, the shape of the seeds, and the design and operation of the mill^8,9^. Compressive and shear forces during grain milling can impact the particle size distribution of flour and the degree of starch damage^10,11^. Changes in starch morphology due to damage during milling, as well as the size of starch granules, can influence the gelatinization of starch^10,12,13^.

In the structure of the endosperm, two main components are distinguished: starch and protein. Starch is embedded in a protein matrix consisting mainly of gluten proteins, which are capable of forming a gluten network^14^. Describing the endosperm structure can be complex. Often, two-dimensional parameters such as the perimeter and surface area of starch granules are used to describe the endosperm structure. However, the dimension of some patterns, such as the contours of biological cells, fractals, and other natural objects, is difficult to describe using Euclidean geometry but can be quantitatively evaluated using measures of complexity^15–17^. Fractal geometry is considered an extension of Euclidean geometry. One of the quantitative measures of complexity is fractal dimension D, which is an exponent of a function where surface profile (S) is dependent on the value of the length of the measuring segment (t). Values of D are between 2 and 3. Structure complexity increases with the value of fractal dimension. Image analysis combined with fractal analysis can be used to quantitatively describe microstructural changes and determine changes of sample properties. This method is used to characterize the microstructure of foods and to clarify the relationship between microstructure and process^18–20^.

Most research on spelt has primarily focused on agronomic and nutritional aspects, while relatively little research has examined the physical and mechanical properties of the grain. Previous studies on the mechanical properties, milling, and grinding of spelt grain did not take into account the context of the endosperm microstructure^21–24^. To minimize damage to the grain in the milling industry, there is a need to develop efficient machines tailored to the threshing of spelt wheat, and their design requires information on the geometrical and mechanical properties of the grain, as well as its internal microstructure. Additionally, assessing the specific energy of milling is essential for forecasting energy needs in an industrial milling process. To the best of our knowledge, the internal structure of the spelt grain endosperm has not been analyzed, especially using numerical image analysis and fractal techniques. With this in mind, this study aims to analyze the endosperm microstructure, physical, thermal properties, and specific milling energy of organic spelt (*Triticum aestivum* ssp. *spelta*) grain and flour.

## Results

### Physical properties of spelt wheat grain

The physical properties of spelt and common wheat grains are presented in Table 1. The hardness indexes (HI) obtained using the SKCS test ranged from 21.4 (Oberkulmer Rotkorn cv. and Zollernspelz cv.) to 71.2 (Schwabenkorn cv.). Based on the HI index, spelt cultivars Oberkulmer Rotkorn, Franckenkorn, and Zollernspelz were classified as soft, while Schwabenkorn spelt and common wheat were classified as hard. The spelt cultivars studied displayed high variability in grain hardness, with greater variability reported for the soft cultivars. The geometric properties and weight of kernels provide information for distinguishing cultivars. The kernel weights of common wheat and spelt grains ranged from 28.2 (Franckenkorn cv.) to 44.5 mg (Oberkulmer Rotkorn cv.). Compared to common wheat, spelt cultivars Oberkulmer Rotkorn and Schwabenkorn had higher grain weight, while Franckenkorn and Zollernspelz had lower grain weight. Oberkulmer Rotkorn spelt was confirmed as having the largest grain length (*L)* 8.35 mm, Schwabenkorn with the largest width (W) 3.52 mm, and common wheat with the largest grain thickness (*T*) 2.97 mm. Of the cultivars studied, the Franckenkorn cultivar had the smallest grain length, width, thickness, and kernel weight. The basic dimensions of the grains of the analyzed spelt cultivars are within the range of changes noted by Kolankowska et al.^24^. Wider grains had a greater mass. The cultivars of spelt and common wheat were differentiated in terms of grain shape. The thinness ratio (R_s_) and circularity factors (R_c_) obtained average values from 1.39 to 1.63 and from 0.78 to 0.85, respectively. With regard to these features, individual cultivars of spelt and common wheat formed three homogeneous groups: Franckenkorn and Zollernspelz, as well as Schwabenkorn and Bombona, while the Oberkulmer Rotkorn cultivar formed a separate group. The grain of Oberkulmer Rotkorn was the most elongated, while those of Schwabenkorn and common wheat were the most spherical.

**Table 1:** Physical properties of spelt and common wheat grain.

Parameter	Oberkulmer Rotkorn			Schwabenkorn			Franckenkorn			Zollernspelz			Common wheat		
	$\overline{X }$	SD	V%	$\overline{X }$	SD	V%	$\overline{X }$	SD	V%	$\overline{X }$	SD	V%	$\overline{X }$	SD	V%
Hardness index, HI (-)	21.4^a^*	17.5	81.7	71.2^c^	17.1	24.0	26.1^b^	16.9	64.7	21.4^a^	14.7	68.7	70.6^c^	16.1	22.8
Kernel weight, KW (mg)	44.5^d^	10.7	24.0	44.5^d^	6.8	15.2	28.2^a^	8.6	30.6	31.3^b^	8.9	28.3	37.4^c^	8.0	21.5
Length, *L* (mm)	8.35^c^	0.56	6.75	7.01^b^	0.36	5.20	6.42^a^	0.50	7.81	6.91^b^	0.61	8.80	6.50^a^	0.34	5.27
Width, *W* (mm)	3.36^d^	0.23	6.99	3.52^e^	0.20	5.64	2.86^a^	0.25	8.86	3.12^b^	0.26	8.33	3.26^c^	0.25	7.74
Thickness, *T* (mm)	2.89^c^	0.21	7.19	2.90^cd^	0.18	6.27	2.61^a^	0.24	8.99	2.81^b^	0.19	6.89	2.97^d^	0.24	7.98
Projection area, *A*_*a*_ (mm^2^)	16.91^c^	1.95	11.55	14.68^b^	1.38	9.41	12.65^a^	1.42	11.23	14.39^b^	1.56	10.83	14.24^b^	1.59	11.14
Projection area, *A*_*b*_ (mm^2^)	20.48^e^	2.35	11.49	18.44^d^	1.65	8.96	14.12^a^	1.60	11.34	16.71^c^	2.22	13.29	15.84^b^	1.69	10.67
Perimeter, *P*_*a*_ (mm)	19.82^c^	1.16	5.84	17.23^b^	0.85	4.94	16.25^a^	1.02	6.26	17.43^b^	1.26	7.22	16.40^a^	0.84	5.14
Perimeter, *P*_*b*_ (mm)	20.46^c^	1.30	6.34	17.89^b^	0.85	4.73	16.66^a^	1.03	6.20	18.21^b^	1.40	7.70	16.58^a^	0.84	5.09
Mean diameter, *D*_*m*_ (mm)	4.32^d^	0.24	5.46	4.15^c^	0.18	4.40	3.63^a^	0.21	5.90	3.92^b^	0.22	5.74	3.98^b^	0.22	5.64
Thinness Ratio, *R*_*s*_ (-)	1.63^c^	0.08	4.75	1.39^a^	0.05	3.35	1.57^b^	0.09	5.51	1.59^b^	0.11	6.83	1.39^a^	0.05	3.67
Circularity factors, *R*_*c*_. (-)	0.78^a^	0.02	2.36	0.85^c^	0.01	1.65	0.80^b^	0.02	2.72	0.79^b^	0.03	3.36	0.85^c^	0.02	1.83

*Values marked with the same letters in rows are not significantly different at p ≤ 0.05; SD, standard deviation; V, coefficient of variation.

### Characteristics of the endosperm microstructure

**Figure 1:**
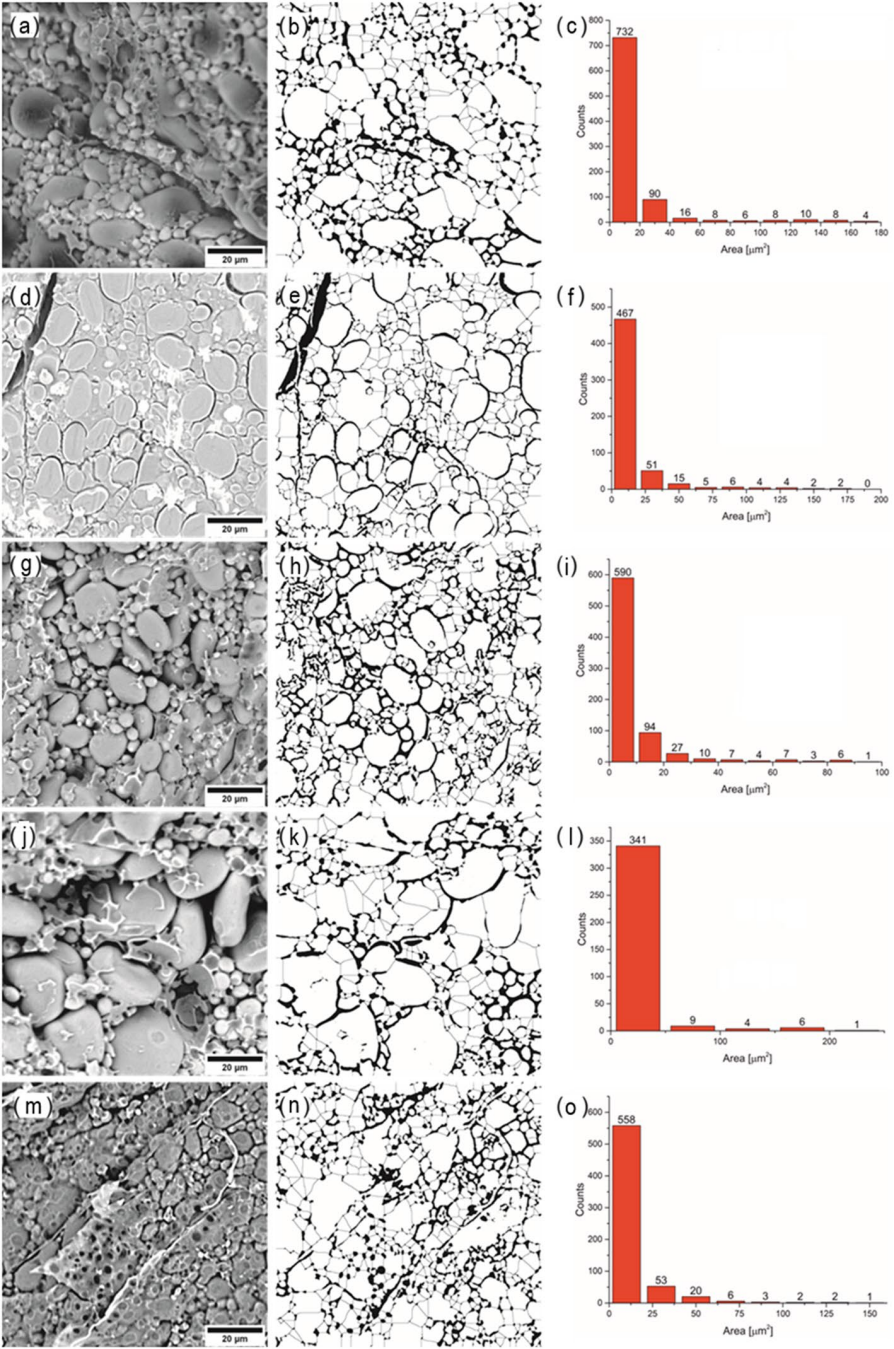
SEM images and corresponding binary maps and histograms of starch of spelt wheat and common wheat: Oberkulmer Rotkorn (**a**–**c**), Schwabenkorn (**d**–**f**), Franckenkorn (**g**–**i**), Zollernspelz (**j**–**l**), Bombona (**m**–**o**).

The microstructure of the starchy endosperm of spelt and common wheat grains is shown in Fig. 1. Observations of the endosperm using SEM revealed clear differences in the microstructure of hard and soft grains. The hard endosperm of Schwabenkorn grains had the most compact structure (Fig. 1d), with well-embedded starch grains and microcracks visible in the protein matrix. The endosperm of the soft grains had a loose structure (Fig. 1a, g, j) with a large amount of undamaged starch grains, but to a lesser extent embedded in the protein matrix. These results are consistent with previous studies of near-isogenic wheat grains varying in hardness^25^. Figure 1c, f, i, l, and o show the number of individual starch size classes (type A, B, and C) determined on the basis of the granule cross-sectional area. From 890 (Oberkulmer Rotkorn) to 366 (Zollernspelz) starch granules were identified on the sections of the endosperm (within the field of observation) of spelt and common wheat. In the endosperm of each cultivar, type C granules (with an area < 20 µm^2^) had the highest fraction, and type A granules (with an area > 177 µm^2^) had the lowest fraction. Among the studied cultivars, the endosperm of the Zollernspelz spelt was distinguished by the largest amount of type A granules, with the largest surface area, reaching a value of up to 510.429 µm^2^ (Fig. 1l). Taking into account the texture of the endosperm, hard cultivars (Schwabenkorn and common wheat) showed a similar percentage of the different types of starch granules, respectively: type A: 0.36 and 0.31%, type B: 15.95 and 13.45%, type C: 83.69 and 86.24%. In contrast, the percentage of individual types of starch granules in the endosperm of soft cultivars (Oberkulmer Rotkorn, Franckenkorn, and Zollernspelz) was not as clear-cut and amounted to: type A: 0.90, 0.04 and 2.46%, type B: 16.85, 9.72, 20.49% and type C: 82.25, 90.24, 77.05%, respectively.

A comparison of the morphological parameters of spelt and common wheat grain endosperm is presented in Table 2. The average cross-sectional area of starch granules ranged from 9.7 µm^2^ (Franckenkorn) to 21.4 µm^2^ (Zollernspelz). The highest percentage of solid phase (starch + protein) was found in Schwabenkorn spelt endosperm at 86.19%, with the lowest in Franckenkorn endosperm at 80.38%. Franckenkorn spelt had the highest degree of coverage, measured by the number of starch particles per one µm^2^ of the tested surface. A higher proportion of large Type-A starch granules resulted in an increased proportion of voids and interphase boundaries in the endosperm. The greater the percentage of these granules, the smaller the percentage of voids and interphase boundaries. These relationships were confirmed by correlation coefficients (p ≤ 0.05), r = − 0.928 and r = − 0.922, respectively.

**Table 2:** Morphological properties of grain endosperm of spelt and common wheat.

Parameter	Oberkulmer Rotkorn	Schwabenkorn	Franckenkorn	Zollernspelz	Common wheat
Mean cross-sectional area of starch granules, (µm^2^)	17.531 ± 33.096*^c^	14.093 ± 26.432^b^	9.658 ± 19.174^a^	21.391 ± 52.142^d^	11.662 ± 22.870^ab^
Min cross-sectional area of starch granules, (µm^2^)	0.009	0.018	0.009	0.009	0.018
Max cross-sectional area of starch granules, (µm^2^)	259.675	236.774	177.004	510.429	321.742
Solid phase share (starch + protein), (%)	84.71 ± 2.57^ab^	86.19 ± 3.03^b^	80.38 ± 2.24^a^	85.46 ± 1.82^b^	83.39 ± 3.19^ab^
Void share, (%)	15.29 ± 0.46^ab^	13.81 ± 0.48^b^	19.62 ± 0.55^a^	14.54 ± 0.31^b^	16.61 ± 0.63^ab^
Share of interphase boundaries, (%)	4.79 ± 0.66^ab^	5.34 ± 0.21^b^	6.96 ± 0.32^c^	4.34 ± 0.46^a^	5.69 ± 1.08^b^
The share of boundaries between starch grains, (%)	1.38 ± 0.20^b^	1.75 ± 0.07^c^	1.6 ± 0.07^bc^	1.07 ± 0.12^a^	1.87 ± 0.37^c^
Coverage rate, (µm^−2^)	0.060 ± 0.010^a^	0.071 ± 0.009^a^	0.104 ± 0.010^b^	0.047 ± 0.010^a^	0.086 ± 0.020^b^
Euler-Poincaré dimension, (χ*10^–3^)	− 0.374 ± 0.058^b^	− 0.479 ± 0.068^a^	− 0.470 ± 0.059^a^	− 0.275 ± 0.055^b^	− 0.510 ± 0.092^a^
Fractal analysis					
Surface anisotropy ratio, Str (-)	0.49 ± 0.03^a^	0.86 ± 0.14^b^	0.91 ± 0.11^b^	0.88 ± 0.09^b^	0.64 ± 0.14^a^
Fractal dimension, D (-)	2.49 ± 0.01^a^	2.59 ± 0.02^b^	2.45 ± 0.03^a^	2.48 ± 0.03^a^	2.58 ± 0.02^b^
Scale length, τ_c_ (µm)	2.22 ± 0.09^c^	1.28 ± 0.12^a^	1.66 ± 0.11^b^	2.98 ± 0.17^d^	1.75 ± 0.30^b^

*Values given as the average values ± standard deviation (SD). Values marked with the same letters in rows are not significantly different at p ≤ 0.05.

The grain cross-section morphology was isotropic, especially for cultivars Schwabenkorn, Franckenkorn, and Zollernspelz, for which the surface anisotropy ratio (Str) was approximately 0.9, indicating a complete lack of distinguished orientation of granule arrangement. The cultivar Oberkulmer Rotkorn had the lowest Str value of 0.49, likely due to a significant predominance of "fine granules". The fractal analysis revealed a monofractal structure for all the cultivars tested. The fractal dimension (D) ranged from 2.45 (Franckenkorn cv.) to 2.59 (Schwabenkorn cv.). Changes in fractal dimension were found to be correlated (p ≤ 0.05) with grain hardness (r = 0.952).

### Mechanical properties and specific milling energy

The mechanical properties and specific milling energy of spelt and common wheat grain are presented in Table 3. During the compression test, the kernels were damaged at an average force (F_r_) ranging from 62.1 N (Franckenkorn cv.) to 88.8 N (common wheat). Soft cultivars of spelt exhibited lower breaking strength of the grain than hard cultivars, but a statistically significant difference was only found between Franckenkorn spelt and Oberkulmer Rotkorn, Schwabenkorn, and common wheat. This indicates that the grain of Franckenkorn spelt had the worst mechanical properties and was more prone to cracking than the grain of the other cultivars. Forces were recorded at displacements (l) ranging from 0.62 mm (common wheat) to 0.80 mm (Schwabenkorn cv.). The grain broke at energy rupture (R_e_) ranging from 19.8 mJ (Franckenkorn cv.) to 31.7 mJ (Schwabenkorn cv.). Spelt grain was characterized by greater variability in grain rupture energy than common wheat, which was attributed to the high variability within a given cultivar.

**Table 3:** Mechanical properties and specific milling energy of spelt and common wheat grain.

Parameter	Oberkulmer Rotkorn	Schwabenkorn	Franckenkorn	Zollernspelz	Common wheat
Rupture force, F_r_ (N)	78.4 ± 22.9^b^*	80.1 ± 27.9^bc^	62.1 ± 16.4^a^	75.4 ± 26.8^ab^	88.8 ± 24.2^b^
Displacement, l (mm)	0.78 ± 0.26^b^	0.80 ± 0.30^b^	0.67 ± 0.22^ab^	0.72 ± 0.26^ab^	0.62 ± 0.17^a^
Rupture energy, R_e_ (mJ)	30.7 ± 17.7^bc^	31.7 ± 21.0^c^	19.8 ± 12.2^a^	25.8 ± 19.1^abc^	21.0 ± 14.9^ab^
Specific milling energy, E_r_ (kJ·kg^-1^)	57.1 ± 5.3^b^	73.0 ± 1.9^c^	47.2 ± 2.0^a^	54.8 ± 2.5^b^	69.5 ± 2.1^c^

*Values given as the average values ± standard deviation (SD). Values marked with the same letters in rows are not significantly different at p ≤ 0.05.

Thicker grain (cultivars: Oberkulmer Rotkorn, Schwabenkorn, and common wheat) required greater force to damage the grain, while grain with greater mass (cultivars: Oberkulmer Rotkorn and Schwabenkorn) required more energy. In relation to soft cultivars of spelt, the lowest breaking strength and energy were shown for the most spherical grains in this group—spelt grains of the Franckenkorn cv. This may be due to the smaller contact surface of round grains with pressing plates, which leads to an increase in the stress gradient within the grain. The average specific energy of milling (E_r_) of grain ranged from 47.2 kJ kg^−1^ (Franckenkorn cv.) to 73.0 kJ kg^−1^ (Schwabenkorn cv.). Soft spelt required significantly less milling energy than Schwabenkorn spelt and common wheat. The average specific energy of milling grains of Franckenkorn, Zollernspelz, and Oberkulmer Rotkorn were approximately 35%, 25%, and 22% smaller, respectively, than hard spelt Schwabenkorn grains.

### Particle size distribution of flour

**Figure 2:**
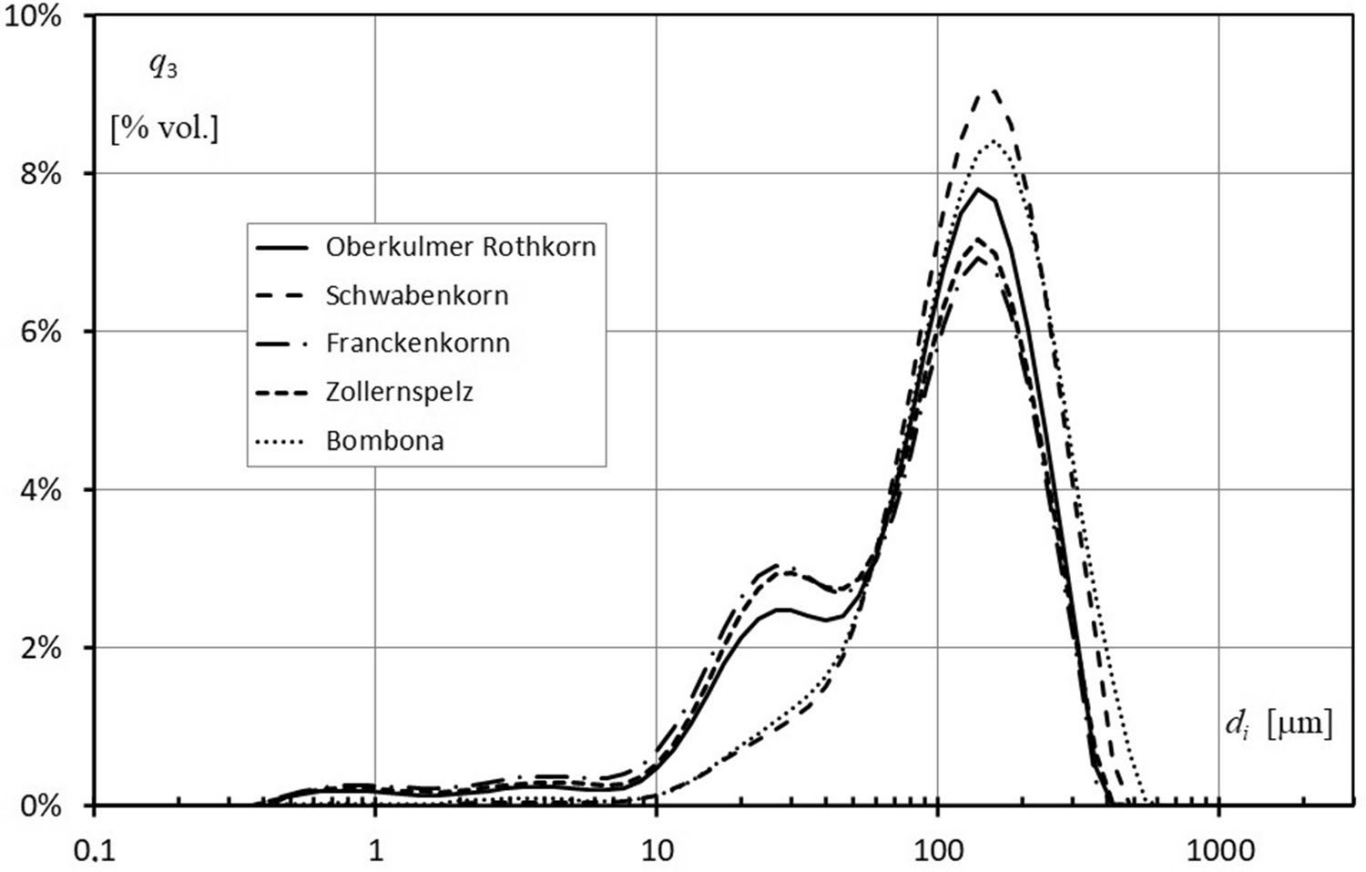
Particle size distribution of spelt and common wheat flour.

The particle size distribution curves of the spelt and common wheat flours are shown in Fig. 2. As predicted, the hard grain flours (Schwabenkorn cv. and common wheat) had a unimodal size distribution, but the very soft grain flours had a bimodal size distribution profile, with a first peak of approximately 25 µm and a second peak of approximately 130 µm. Each of the tested flours also had a trace fraction of about 0.8 and 3 µm. The studied cultivars of spelt formed two groups that were homogeneous in terms of grain hardness and thus gave two different grinding patterns. Hard cultivars, such as Schwabenkorn and common wheat, ware characterized by a trace proportion of dusty fractions, while soft spelt contains about three times the proportion of these fractions. The average particle size of flours (d_f_) depended on grain hardness and ranged from 87.5 µm (Franckenkorn cv.) to 115.5 µm (common wheat) (Table 4). The average particle sizes of the flours from hard grains of Schwabenkorn spelt and common wheat were larger than those obtained from soft grains and were determined in the range of 116.5 and 115.5 µm, respectively. The flours produced from the soft spelt grain cultivars of Oberkulmer Rotkorn, Franckenkorn, and Zollernspelz had a greater amount of fine particles than other flours. The values of d(0.1) for these flours were 17.9, 13.7, and 16.1 µm, respectively. Additionally, these cultivars had a wider relative width of distribution (SPAN) which implies that their flour particles were less uniform in size.

**Table 4:** Physical properties of spelt and common wheat flour.

Parameter	Oberkulmer Rotkorn	Schwabenkorn	Franckenkorn	Zollernspelz	Common wheat
Average size of the particles, *d*_*f*_ (µm)	96.5 ± 1.0^c^*	116.5 ± 0.4^d^	87.5 ± 0.8^a^	90.5 ± 1.0^b^	115.5 ± 0.8^d^
*d(0.1)* (µm)	17.9 ± 0.6^c^	42.8 ± 0.5^e^	13.7 ± 0.4^a^	16.1 ± 0.4^b^	40.1 ± 1.4^d^
*d(0.5)* (µm)	97.9 ± 1.6^c^	123.5 ± 0.9^d^	85.0 ± 1.3^a^	89.6 ± 1.7^b^	126.3 ± 1.5^d^
*d(0.9)* (µm)	212.1 ± 2.6^c^	247.3 ± 1.6^c^	204.1 ± 1.3^a^	209.1 ± 3.1^ab^	266.6 ± 5.1^d^
SPAN (-)	1.98 ± 0.01^c^	1.66 ± 0.02^a^	2.24 ± 0.03^e^	2.15 ± 0.02^d^	1.79 ± 0.02^b^
Damaged Starch (%)	3.84 ± 0.06^ab^	6.14 ± 0.14^d^	3.69 ± 0.08^a^	4.11 ± 0.10^b^	5.00 ± 0.14^c^
DSC of flour					
*T*_*o*_ (°C)	59.4 ± 0.3^c^	56.3 ± 0.2^a^	57.9 ± 0.8^b^	58.4 ± 0.5^b^	61.0 ± 0.1^d^
*T*_*p*_ (°C)	64.1 ± 0.2^d^	62.3 ± 0.1^a^	63.8 ± 0.1^c^	63.2 ± 0.2^b^	65.1 ± 0.2^e^
*T*_*e*_ (°C)	68.8 ± 0.6^ab^	68.0 ± 1.2^a^	68.4 ± 0.2^a^	68.4 ± 0.4^a^	69.8 ± 0.3^b^
*ΔT* (°C)	9.4 ± 0.4^ab^	11.7 ± 1.0^c^	10.6 ± 0.8^bc^	10.0 ± 0.8^ab^	8.8 ± 0.3^a^
*ΔH* (J g^−1^)	5.3 ± 0.1^a^	5.8 ± 0.4^ab^	6.3 ± 0.5^b^	5.6 ± 0.7^ab^	5.4 ± 0.3^a^

*Values given as the average values ± standard deviation (SD). Values marked with the same letters in rows are not significantly different at p ≤ 0.05.

### Starch damage of flour

The percentage of damaged starch as a result of grain milling varied from 3.69% (Franckenkorn cv.) to 6.14% (Schwabenkorn cv.) (Table 4). The flours of Schwabenkorn and common wheat showed the highest degree of starch damage, 6.14% and 5.00% respectively. During milling, starch granules in grains with a hard texture were significantly damaged. A reduction of starch damage was observed in soft grains, as shown in Fig. 1a, g, j and confirmed by the values of the share of boundaries between starch grains. The degree of starch damage of soft cultivars increased when the number of type A and type C granules in the endosperm increased (Fig. 1c, i, l and Table 4).

### Thermal properties of flour

Thermal properties of investigated spelt wheat materials are shown in Table 3. The initial temperature of gelatinization and the peak temperature differed significantly between the tested cultivars of spelt and common wheat. The transition temperatures to gelatinization: onset temperature (*T*_*o*_), peak temperature (*T*_*p*_) and end temperature (*T*_*c*_) recorded for common wheat were the highest, respectively: 61.0, 65.1, 69.8 °C. The highest values of temperatures: *T*_*o*_, *T*_*p*_, and *T*_*c*_ of spelt flour were recorded for spelt cv. Oberkulmer Rotkorn and the smallest for cv. Schwabenkorn, respectively: 59.1, 64.1, 68.8 °C and 56.3, 62.3, 68.0 °C. The lower values of *T*_*o*_, *T*_*p*_, and T_c_ of spelt flours indicate that a lower temperature is needed to gelatinize spelt starch than to gelatinize common wheat starch. The gelatinization temperature range (*ΔT*) was significantly (p ≤ 0.05) minimal in common wheat flour (8.8 °C) and maximum in Schwabenkorn flour (11.7 °C). The increased *ΔT* compared to common wheat indicates a lower homogeneity of the starch granules in Schwabenkorn (11.7 °C), Franckenkorn (10.6 °C) and Zollernspelz (10.0 °C) flours before gelatinization. The change in gelatinization enthalpy (ΔH) of spelt and common wheat ranged from 5.3 J g^−1^ (Oberkulmer Rotkorn cv.) to 6.3 J g^−1^ (Franckenkorn cv.). A significant difference (p ≤ 0.05) in the enthalpy change was found between spelt cvs. Franckenkorn, and Oberkulmer Rotkorn, as well as common wheat.

## Discussion

The results of the study indicate that spelt grains of the tested cultivars exhibit significant variations in their dimensional properties and shape. The genetic variation of each cultivar is thought to be the main cause of these differences in geometrical dimensions of the grain^7,26^. The analysis of 10 geometrical features of the grain revealed that the greatest variability was found in relation to the projection area A_a_ and A_b_, with the smallest variability observed in relation to the circularity coefficient R_c_. This was consistent across all cultivars studied. These findings are in line with previous research^7,21,27^. Person's correlation showed that the fractal dimension (D) had a negative correlation (p ≤ 0.05) with thinness ratio *R*_*s*_ (r = − 0.915) and a positive correlation with circularity factors *R*_*c*_ (r = − 0.901). A correlation between D and *R*_*s*_ or *R*_*c*_ suggests that the fractal dimension is able to capture information about the shape of the grains.

Observations of SEM images on samples of spelt wheat cultivars classified as hard and soft by the SKCS confirmed the degrees of variation in endosperm structure. These results are consistent with studies of near-isogenic wheat grains varying in hardness^25^. In the endosperm of each cultivar, type C granules had the highest fraction, and type A granules had the lowest fraction. It is important to note that, unlike isolated starch, when analyzing an image of a cross-section of the endosperm, one can only see the granules that are located within the imaged section of the endosperm. Within the imaged section of the endosperm, hard varieties showed a similar percentage of different types of starch granules, as opposed to soft varieties. Previous studies have shown that the size distribution of wheat starch granules varies by genotype, environmental conditions during growth, and their location within the grain^28,29^. The different proportions of individual types of starch granules in flours made from the grain of soft spelt cultivars may result in various functionalities of these flours. Shang et al.^12^ report that starch granule size affects flour functionality and final quality. The proportion of voids and interfacial boundaries in the endosperm of spelt was mainly related to the number of large A-type granules—the higher their proportion, the lower the proportion of voids and interfacial boundaries. An increased proportion of voids and interfacial boundaries in the endosperm led to a reduction in starch damage during milling.

The analysis of the degree of structure development revealed the monofractal structure of all the cultivars studied. Hard cultivars showed on average 4.45% higher values of dimension D compared to soft spelt cultivars. Harder kernels may have a more complex shape than softer kernels, resulting in a higher fractal dimension^30^. According to Turnbull et al.^31^, analysis of SEM images shows that endosperm surfaces of hard grains exhibit a higher degree of isotropy compared to soft grains.

The studied varieties of spelt were characterized by similar values of forces and energy during grain breakage as those studied by Żuk-Gołaszewska et al.^22^. Soft spelt cultivars showed lower grain rupture force than hard cultivars. The mechanical strength of the grain can have a significant impact on grain damage. Among all the spelt cultivars analyzed, the soft spelt Franckenkorn cultivar was the most susceptible to mechanical kernel damage. The kernels of this cultivar broke under a force 30% less than that of common wheat grains. This may be due to the anatomical structure of the grain. The Franckenkorn cultivar of spelt had kernels with the smallest length, width, thickness, and weight and had the smallest share of solid phase and average cross-sectional area of starch granules in the endosperm. It is known that the mechanical properties of wheat grain depend, among other things, on the anatomical differences between peripheral tissues, starch endosperm, and the chemical composition and distribution of individual elements within the structure. The strength of the endosperm is determined by protein content, starch granule size, and adhesion between starch granules and the protein matrix^25,32–37^. The evaluation of mechanical properties of grain is closely related to the milling process and serves as a basis for predicting energy demand in this process^38^.

The grain hardness of spelt cultivars was a property that differentiated specific milling energy, the particle size distribution of flour and the rate of starch damage. Previous studies have shown that the specific energy required for milling depends on the method used, the moisture content of the grain, the kernel hardness, and the desired fineness of the final product^23,39^. Our study found similar relationships. The average specific energy required for milling had a significant correlation (p ≤ 0.05) with grain hardness (r = 0.906) and the average size of the flour particles (r = 0.979). Additionally, we found that the average specific energy required for milling the soft grains Franckenkorn, Zollernspelz, and Oberkulmer Rotkorn was approximately 35%, 25%, and 22% lower, respectively, compared to the hard spelt Schwabenkorn grains. However, it should be noted that the energy consumption for milling the soft grains was found to be higher compared to the values reported by Świeca et al.^23^. Differences in particle size distribution are clearly related to wheat grain hardness^40–42^. Hard grain flours (Schwabenkorn and Bombona) had a unimodal size distribution, while soft grain flours had a bimodal particle size distribution profile with a higher proportion of fine particles. Flours from soft spelt genotypes had a higher percentage of fine particles compared to flours from hard grain, as indicated by d(0.1) values. The hardness index was correlated (p ≤ 0.05) with d(0.1), d(0.5), d(0.9), and SPAN, respectively: r = 0.983, r = 0.953, r = 0.952 and r = − 0.879. The negative correlation between grain hardness and SPAN indicates less uniformity in the size of particles in flours obtained from soft spelt grain. The particle size of wheat flour affects its functional properties as well as the rheological properties of dough, thereby affecting the quality of the final products^43,44^.

The greatest degree of starch damage was found in flours obtained from hard wheat. The degree of starch damage in flour had a significant correlation (p ≤ 0.05) with grain hardness (r = 0.899). This is consistent with the results of previous studies^29,35^, which have shown that the degree of starch damage is related to the forces of adhesion between the starch granules and the protein matrix. The reduction of adhesion force between the starch granules and the protein matrix leads to a decrease in starch damage in soft wheat. The protein matrix and cell walls in the wheat grain can protect the starch granules from structural degradation during milling. An adequate content of damaged starch in the flour is a key factor that affects the quality of bread, as it improves water absorption capacity, dough viscosity, and facilitates yeast fermentation^45^. The changes in the fractal dimension of the endosperm microstructure images were correlated (p ≤ 0.05) with specific milling energy, average size of the particles of flour, and starch damage rate (respectively: r = 0.991, 0.993, 0.923). A higher fractal dimension indicates a more complex microstructure. A significant correlation between the fractal dimension of the endosperm microstructure and specific milling energy, average size of the particles of flour, and starch damage rate suggests that the complexity of the endosperm microstructure plays a role in determining these properties.

Gelatinization of starch is an endothermic process during which, under certain conditions of heat and humidity, the starch changes from a native semi-crystalline state to an amorphous structure^45^. The highest ΔH of the Franckenkorn flour suggests that the greatest heat energy is needed to gelatinize its starch, i.e. to achieve the most desirable baking characteristics, compared to other flours. The gelatinization temperature of starch granules in flour particles is higher than in the case of starch granules isolated from them, and non-starch components in flour may affect the starch gelatinization temperature^10^. The highest values of gelatinization enthalpy are observed for Schwabenkorn, Franckenkorn, Zollernspelz and the same cultivars exhibit the lowest temperatures of DSC peaks. This phenomenon may be caused by the lowest average molar masses of starch molecular chains. Additionally, flour from these three cultivars shows the highest values of onset and end temperature difference, which corresponds to high polydispersity of molecular chains. In the presented research, starch damage is not correlated with the thermal parameters of the gelatinization process. This is in direct contradiction with the results obtained by other authors^46,47^. The cause of this may be the protective role of the protein matrix during the gelatinization process in flour^48^. Results presented in earlier works mainly focus on gelatinization parameters for isolated starch. In those works, peak temperatures are around 70 °C or higher, and the process enthalpy has values of 11–13 J g^−1^. In the presented work, both the value of gelatinization enthalpy is lower. This would be consistent with the protective role of the protein matrix, as the competitive process of protein denaturation exhibits lower enthalpy^49^. The thermal parameters of water flour reaction are not correlated with parameters of mathematical endosperm structure analysis and therefore require additional testing.

## Conclusion

In conclusion, this study has shown that there are significant variations in the dimensional properties, shape, and microstructure of the endosperm of spelt grains. The soft spelt Franckenkorn cultivar stood out with the smallest grain size and weight, and it also showed the greatest differences in microstructural properties when compared to the other investigated spelt cultivars. All the cultivars tested have a monofractal structure, which implies that a single fractal dimension is sufficient to describe their structural complexity. This dimension is correlated to kernel hardness, specific milling energy, the particle size distribution of flour, and the starch damage rate. The findings of this study provide useful theoretical guidance for understanding the relationship between the structure of the spelt endosperm and its physical properties. They can also contribute to the prediction of flour quality and the design of machinery and equipment used in spelt grain processing. Furthermore, the results suggest that fractal analysis may be a valuable tool for further evaluations of milling processes.

## Materials and methods

### Materials

Experimental material consisted of four cultivars of spelt wheat: Oberkulmer Rothkorn, Schwabenkorn, Franckenkorn, Zollernspelz, and common wheat cv. Bombona. Grain samples (approx. 4 kg of each sample) were harvested from North-Eastern Poland (53°19′30″ N, 19°28′38″ E). The harvested spelt grains were subjected to dehulling using a BK 1100 huller from LFMR SA in Poland. The grain samples were placed in plastic bags and stored at 6 °C until they were used further. To determine the moisture content of the kernels, AACC Method 44-15.02^50^ was employed. The hardness index and kernel weight of 300 kernels were assessed using the Single Kernel Characterization System (SKCS) type 4100 from Perten Instruments North America Inc. in Reno, USA, as per AACC Method 55-31.01^50^.

### Geometrical properties

From the seed mass, 110 kernels of each variety were randomly selected and scanned with a Plustek Optic Pro ST 24 flatbed scanner at a resolution of 1200 dpi. The scanning was performed for two projections of grains: projection *a*—grains arranged sideways and projection *b*—grains arranged with the furrow down. Based on the images obtained in this way, the measurements of the geometrical parameters of the seeds were performed using the ImageJ program (v. 1.51 h, Laboratory for Optical and Computational Instrumentation, Madison, WI, USA). The examined geometrical features of seeds included: length (*L*), width (*W*), thickness (*T*), projection area (*A*_*a*_ and *A*_*b*_), projection perimeter (*P*_*a*_ and *P*_*b*_). Geometric projection equivalent diameter (*D*_*m*_), thinness ratio (*R*_*s*_) and coefficients of circularity (*R*_*c*_) of kernels were calculated using Eqs. (1)–(3)^51^.

1 $$Dm=\sqrt[3]{LWT}$$

2 $${R}_{s }= \frac{{P}_{b}^{2}}{4\pi {A}_{b}}$$

3 $${R}_{c}=\frac{2\sqrt{\pi {A}_{b}}}{{P}_{b}}$$

### Mechanical properties

The mechanical properties of individual kernels were determined by a quasi-static compression test using a Mecmesin Limited, Slinfold, UK testing machine, equipped with a 0.5 kN load head. Single kernels (randomly selected from a grain batch) were placed on the bottom plate with the furrow down and compressed at the speed^21^ of 5 mm min^−1^. Force values and head displacement were recorded on a PC connected to the device, equipped with the EmperorTM Force Testing System software (Mecmesin Ltd., Slinfold, UK). The mechanical behavior of the grain was expressed as the maximum rupture force (F_r_), the force required to damage a kernel, and the rupture energy(R_e_), the work required to damage a kernel. The test was carried out on 30 randomly selected kernels of each cultivar with a moisture content of 12.4 ± 0.2% w.b.

### Morphological properties of grain endosperm

The internal structure was assessed on the cross-sections of the kernels by scanning electron microscopy (SEM) using the Thermo Scientific ™ Phenom ™ ProX G6 Desktop SEM instrument. The analysis of the internal morphology of the grains (endosperm) was based on the segmentation of binary SEM images using the "watershed" method and the Minkowski analysis^52^.The use of Minkowski's functionals (*V, S,* $\upchi$) in the analysis of the grain morphology made it possible to determine: the share of the solid phase (*V*) and voids, the relative share of interfacial boundaries (*S*) and the Euler-Poincaré dimension ($\upchi$). The quoted Minkowski functions in relation to the binary maps presented in Fig. 1 (middle column) are described by Eqs. (4)–(6).

4 $$V=\frac{{N}_{w}}{N} \cdot 100\%$$

5 $$S=\frac{{N}_{bound}}{N}\cdot 100\%$$

6 $$\upchi =\frac{{C}_{w}-{C}_{b}}{N}$$

in which: *N*—number of pixels, *N*_*w*_—the number of white pixels, *N*_*bound*_—the number of white and black pixel borders, *(C*_*w*_*−C*_*b*_*)*—the difference between the number of white and black areas^53^. The criterion for the division of starch granules into types A, B and C were the mean diameter of granules from the trimodal distribution^13,28,54^ and the corresponding surface area of a circle with the diameter, where: type—A with a diameter > 15 μm, (> 177 μm^2^), type B with average from 5 to 15 μm (from 20 to 177 μm^2^) and type C with a diameter < 5 μm (< 20 μm^2^).

### Image analysis and fractal characterization

Previous works demonstrated that SEM images can be treated in the same way as AFM data in order to get the following characteristics of surface patterning: surface anisotropy ratio, fractal dimension and scale length^55^.

**Figure 3:**
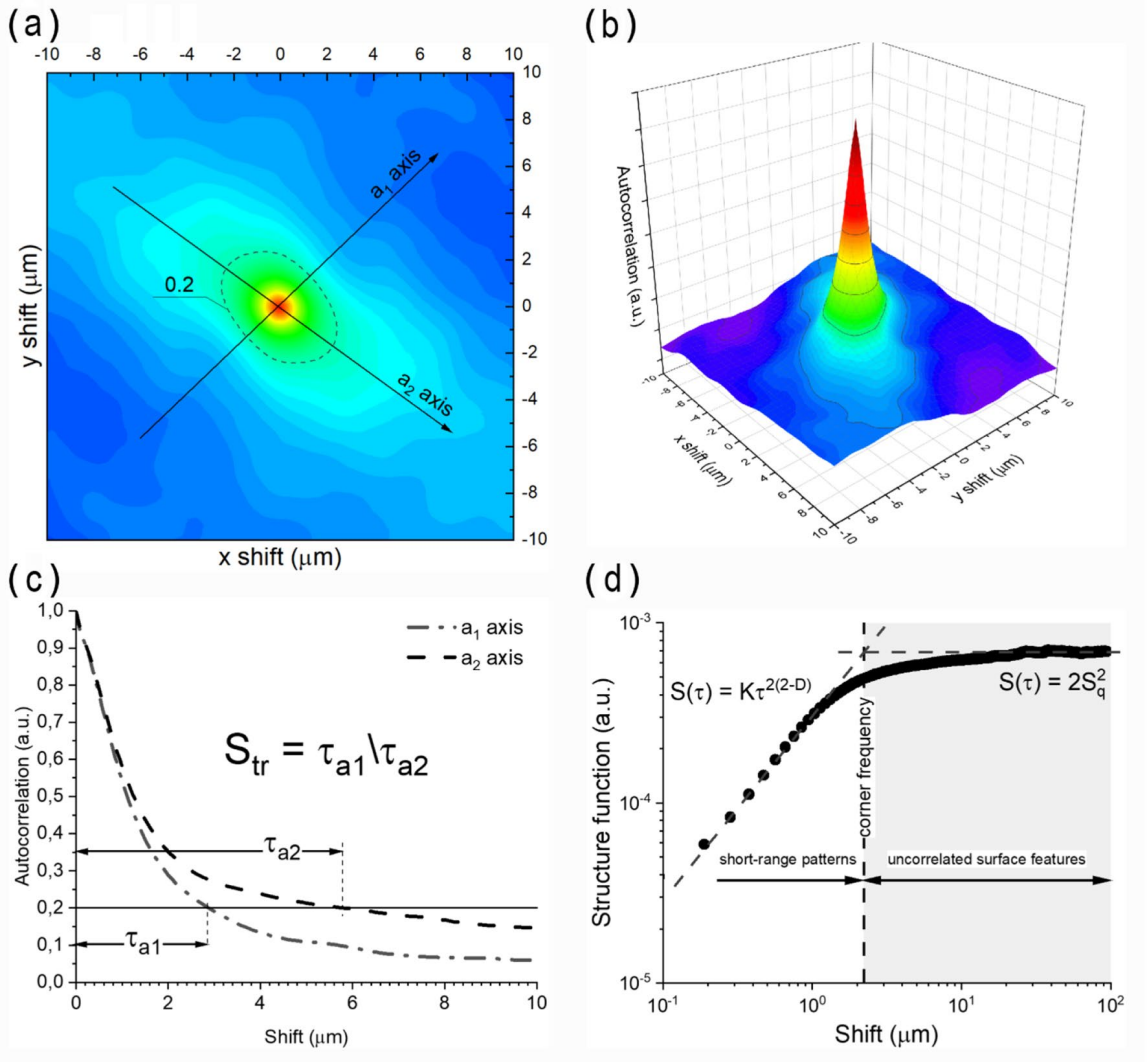
Example plots of autocorrelation and structure functions derived from SEM image shown in Fig. 1a: (**a**) plane projection of 2-dimensional autocorrelation function drawn with the axes of the fastest and slowest autocorrelation decays down to 0.2 value (a_1_ and a_2_, respectively), (**b**) 3-dimensional autocorrelation function (main peak), (**c**) profiles of the autocorrelation function along a_1_ and a_2_ axes, (**d**) log–log plot of the structure function.

Directionality of predominant surface patterns is reflected in the surface anisotropy ratio S_tr_ defined according to (Fig. 3a) as:

7 $$S_{tr} = \left. {\frac{{\tau_{a1} }}{{\tau_{a2} }}} \right|_{R = 1 \to 0.2}$$

where: *a*_*1*_ and *a*_*2*_ are the axes of the slowest and fastest decays of the autocorrelation function, respectively.

In contrast to scale-dependent anisotropy, fractal approach results in scale-invariant measures estimated from the structure function. Almqvist^56^ demonstrated that the structure function reveals allometric-like dependence on the scale length τ:

8 $$S\left( \tau \right) = K\tau^{{2\left( {2 - D} \right)}}$$

where: D is the fractal dimension, and K is the scaling factor. At some threshold the structure function sharply goes flat at 2S_q_^2^ level, which is referred to as the corner frequency (shown in Fig. 3b).

Apart from that, overlapping objects in SEM images can be split into independent entities using the watershed algorithm. Similar to grain analysis, topography patterns are resolved depending on their position within layers of different elevation, which allows for computation of statistical parameters concerning the relative contribution of hills and valleys. Example results are shown in Fig. 3c, d.

### Specific milling energy

The milling was performed on a laboratory scale. Before grinding, the grain of each variety was moistened to 15% humidity. The 125 g grain samples were weighed with an accuracy of ± 10 mg on a WLC 2/A1 electronic balance (Radwag®, Poland), and then milled in a Quadrumat Junior roller mill (Brabender®, Germany). The sifting sieve was removed from the mill in order to obtain middlings. The seven replicates of middlings from each wheat cultivar were separated with a sieve shaker (Analysette 3® Fritsch, Germany) equipped with 200 µm sieve, followed by a collection pan. The sample was shaken for 10 min (amplitude of the vibration was 1.5 mm). The fraction passing through the 200 µm sieve was treated as extracted flour. Specific milling energy E_r_ (kJ kg^−1^) was calculated with the following formula (9):

9 $${E}_{r }= \frac{{E}_{c}- {E}_{s}}{{m}_{g}}$$

where: *E*_*c*_—total energy consumed by the mill; *E*_*s*_—energy required for initiating the motion of ground particles (*E*_*s*_ was calculated by multiplying active power in idle mode by milling time); *m*_*g*_—mass of milled sample (kg).

### Particle size analysis and damaged starch of flour

A Malvern Mastersizer 2000 particle size analyzer (version 5.22, Malvern Instruments Ltd, Malvern, UK) in wet dispersion mode was used to measure the size of the flour particles. The average particle size of the flours was calculated as the sum of the of particle sizes (*d*_*i*_) multiplied by volume fractions (*φ*_*i*_) of the fraction *"i": d*_*avg*_ = SUM(*φ*_*i*_* · d*_*i*_). The particle sizes *d (0.1), d (0.5)* and *d (0.9)* (µm) were also determined, which correspond to the minimum, median and maximum particle size of the sieving, respectively 10%, 50% and 90% of the particles. The relative width of the distribution (SPAN) was defined using formula (10).

10 $$SPAN= \frac{d(0.9)-d(0.1)}{d(0.5)}$$

Damaged Starch in samples of flours were determined according to AACC Method 76-31.01^50^.

### Differential scanning calorimetry (DSC)

A DSC 204 F1 Phoenix®—NETZSCH (Germany) differential scanning calorimeter was used to determine the starch gelatinization properties. The spelt flour was weighed into an aluminum crucible (about 9 mg). Then, using a pipette, distilled water was added in a mass ratio of 1:2 (flour/water). The crucibles were sealed and allowed to stabilize at room temperature overnight prior to analysis. The crucibles was heated from 20 to 90 °C at a rate of 5 °C/min. An empty crucible was used as a reference. Onset temperature (*T*_*o*_), peak temperature (*T*_*p*_), end temperature (*T*_*c*_), temperature difference (*ΔT*), where: (*ΔT*) = *T*_*c*_*−T*_*o*_ and the gelatinization enthalpy (*ΔH*), were determined on the basis of the endotherm of starch gelatinization using the built-in NETZSCH Proteus—Thermal Analysis- v. 5.2.1 software.

### Statistical analysis

The test results were statistically processed using descriptive statistical methods, parametric and nonparametric tests using the STATISTICA® computer program for Windows v. 13.3 (TIBCO, Paolo Alto, USA). A one-way analysis of variance (ANOVA) was performed for the variant. The significance of differences between the means was assessed using Duncan’s test. When the empirical data was not normally distributed, the Kruskal–Wallis test was used to determine significant differences between the analyzed parameters. Coefficients of variation (CV) were calculated as the ratio of the standard deviation to the mean. The calculations were made at the significance level p ≤ 0.05. Pearson correlation analysis was carried out for all analyzed variables.

## Data Availability

The datasets generated during and/or analysed during the current study are available from the corresponding author on reasonable request.
